# Epigenetic Plasticity Enables CNS-Trafficking of EBV-infected B Lymphocytes

**DOI:** 10.1371/journal.ppat.1009618

**Published:** 2021-06-09

**Authors:** Samantha S. Soldan, Chenhe Su, R. Jason Lamontagne, Nicholas Grams, Fang Lu, Yue Zhang, James D. Gesualdi, Drew M. Frase, Lois E. Tolvinski, Kayla Martin, Troy E. Messick, Jonathan T. Fingerut, Ekaterina Koltsova, Andrew Kossenkov, Paul M. Lieberman

**Affiliations:** 1 The Wistar Institute, Philadelphia, Pennsylvania, United States of America; 2 The University of Pennsylvania School of Medicine, Philadelphia, Pennsylvania, United States of America; 3 Saint Joseph’s University, Philadelphia, Pennsylvania, United States of America; 4 Cedars-Sinai Medical Center, Los Angeles, California, United States of America; Duke University Medical Center, UNITED STATES

## Abstract

Subpopulations of B-lymphocytes traffic to different sites and organs to provide diverse and tissue-specific functions. Here, we provide evidence that epigenetic differences confer a neuroinvasive phenotype. An EBV+ B cell lymphoma cell line (M14) with low frequency trafficking to the CNS was neuroadapted to generate a highly neuroinvasive B-cell population (MUN14). MUN14 B cells efficiently infiltrated the CNS within one week and produced neurological pathologies. We compared the gene expression profiles of viral and cellular genes using RNA-Seq and identified one viral (EBNA1) and several cellular gene candidates, including secreted phosphoprotein 1/osteopontin (SPP1/OPN), neuron navigator 3 (NAV3), CXCR4, and germinal center-associated signaling and motility protein (GCSAM) that were selectively upregulated in MUN14. ATAC-Seq and ChIP-qPCR revealed that these gene expression changes correlated with epigenetic changes at gene regulatory elements. The neuroinvasive phenotype could be attenuated with a neutralizing antibody to OPN, confirming the functional role of this protein in trafficking EBV+ B cells to the CNS. These studies indicate that B-cell trafficking to the CNS can be acquired by epigenetic adaptations and provide a new model to study B-cell neuroinvasion associated CNS lymphoma and autoimmune disease of the CNS, including multiple sclerosis (MS).

## Author summary

Trafficking of pathogenic B-cells to the CNS can drive CNS lymphoma and multiple sclerosis (MS). Here, we show that EBV+ B-cells can undergo an epigenetic switch conferring a neuroinvasive phenotype with upregulation of SPP1/Osteopontin, which when blocked reduces CNS penetrance.

## Introduction

Epstein-Barr virus (EBV) is a near ubiquitous gamma-herpesvirus that establishes latent infection in long-lived B-lymphocytes and is associated with diverse human disease, including cancer and auto-immune disorders [1–3]. EBV is typically transmitted orally by episodic shedding of virus from the oropharynx of the carrier via salivary contact with permissive epithelial cells [4,5]. The virus transiently infects epithelial and lymphoid cells in the Waldeyer’s ring of the oropharynx but undergoes a stable transformative infection in B cells expressing CD21, the major cellular receptor for EBV [6–8]. The virus induces developmental changes in resting B-cells that mimic germinal center reactions and ultimately memory B-cell phenotype (IgD-CD27+ and IgD+CD27+) where the virus establishes life-long infection [9,10]. During this B-cell transformation process, both EBV and cellular genomes undergo varying degrees of epigenetic modification, including silencing of many viral genes necessary to establish stable latent infection [11–13]. Spontaneous reactivation from latency can release newly infectious virus, and this reaction mimics memory B-cell terminal differentiation to plasma cells and cell death [14,15]. In healthy carriers, EBV is found in only ~1 per million circulating B-cells, but virus may reside in B-cells at diverse stages of differentiation in lymphoid organs [16].

While most EBV infections lead to the establishment of benign latent infection, EBV can also be the causative agent of a diverse spectrum of lymphoid and epithelial malignancies, including Burkitt’s and Hodgkin’s lymphomas, NK/T cell lymphomas, and lymphoepithelial nasopharyngeal and gastric carcinomas [17–19]. EBV-associated lymphoproliferative disease and primary central nervous system lymphoma (PCNSL) can occur during immunosuppression [19]. In addition, a growing body of epidemiological, virological, and immunological evidence supports a major role for EBV in autoimmune disorders, including, multiple sclerosis (MS), systemic lupus erythematosus (SLE), rheumatoid arthritis (RA), juvenile idiopathic arthritis, inflammatory bowel disease, celiac disease, and type 1 diabetes [20].

Central nervous system (CNS) complications from EBV infection have long been appreciated (reviewed in [21] and the earliest descriptions of Burkitt’s lymphoma suggest that most patients (21/25 in an early autopsy series) had tumors involving the brain or meninges [22]. Other malignancies involving EBV infected B cells in the CNS include PCNSL, a primary intracranial tumor of B-cell origin that accounts for 1% of all lymphomas and 3–5% of primary brain tumors [23,24] and is commonly, though not exclusively, observed in patients who are immunocompromised. HIV infection is the highest risk factor for PCNSL, which accounts for 15% of HIV-associated lymphomas [25]. As with all masses in the central nervous system (CNS), the location of PCNSL lesions determines the clinical presentation [26]. Because of the depth of the tumor, PCNSLs are not typically amenable to surgical resection and the clinical outcomes of PCNSL and other EBV-associated malignancies that have metastasized to the CNS are poor [25,26].

In addition to the CNS malignancies associated with EBV, growing evidence supports an association between EBV and autoimmune disorders affecting the CNS, including MS and SLE. MS is the most prevalent disabling demyelinating disease of the CNS, affecting an estimated one million individuals in the United States alone [27–31]. The etiology of MS is thought to be multifactorial with genetic, immunological, and environmental factors contributing collectively to MS susceptibility [32–34]. Among these, Epstein-Barr virus (EBV) infection is the most significant viral etiological risk factor [35–39]. MS patients are almost always EBV positive, and a history of EBV-positive infectious mononucleosis (IM) increases risk of MS by ~40 fold [40,41]. Elevated antibodies to the EBV latency-associated nuclear antigen 1 (EBNA1) are frequently observed prior to the onset of neurological symptoms [39,40,42–46]. In addition, evidence for EBV infection in brain-infiltrating B cells in MS-patient brain lesions has been reported [47–49], although conflicting reports fail to find EBV infected cells in CNS lesions [50,51] including a recent study of the B-cell transcriptome in B-cells obtained from the CSF and peripheral blood of MS patients was devoid of transcripts of human viruses, including EBV [52]. Therefore, how EBV contributes to MS pathogenesis is not well understood. Numerous studies have described aberrant immune responses to EBNA1 in patients with MS [53–55]. EBV has also been proposed to promote the survival of auto-reactive B cells that produce oligoclonal antibodies in cerebrospinal fluid of MS patients, as well as promote the survival and penetration of infected B cells into the CNS to drive MS pathogenesis [35,56,57]. The importance of B cells in MS pathology has been underscored by the robust efficacy of anti-CD20 B cell depletion therapies, such as rituximab, ofatumumab, and ocrelizumab [58,59], in treating patients with relapsing remitting and primary progressive MS. Other treatment strategies targeting pathogenic B cells, including the purine analog cladribine, which reduces the population of CD19+ B cells, have been successfully implemented in MS [60]. Moreover, recent studies showing promising clinical outcomes using adaptive transfer of autologous EBV-specific T cells support a pathogenic role of EBV+ B cells in the pathogenesis of MS [61].

CNS trafficking of EBV+ B cells is a critical step in the development of PCNSL and also plays an important role in the pathogenesis of MS. However, viral and cellular determinants that promote neuroinvasion of EBV+ B cells into the CNS are not well defined. To better understand EBV-encoded and cellular factors that promote CNS penetration of EBV+ B cells, we developed a mouse model to study the infiltration of EBV immortalized B cells in the CNS using a neuroadapted, EBV+ B lymphoma line (MUN14) with an increased neuroinvasive phenotype compared to the parent line (M14). We investigate the mechanisms underlying B-cell neurovinasion and find that sustained epigenetic changes correspond to differential expression of cellular genes implicated in B-cell trafficking and function. We demonstrate that one of these differentially regulated genes, SPP1/osteopontin (OPN), functions to promote CNS trafficking and neuroinvasion of EBV+ B-cells.

## Results

### Generation of an EBV+ BL cell line with enhanced CNS trafficking

**Fig 1:**
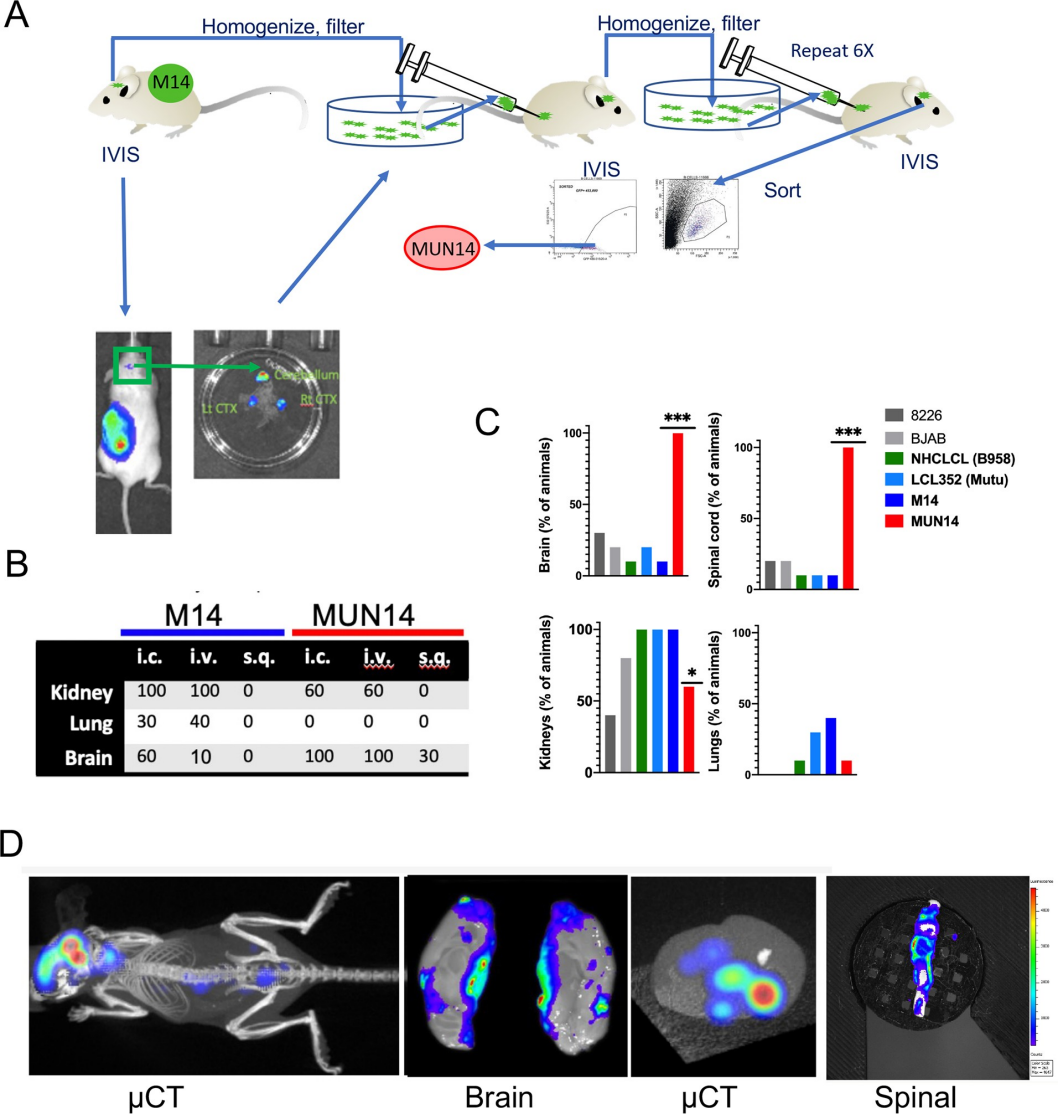
Neuroadaptation of an EBV+ B cell lymphoma line (MUN14) from parental Mutu I (M14). **A.** The neuroadapted MUN14 line was obtained by serially passaging M14 cells that metastasized to the brain, through seven mice. These cells were purified by sorting for GFP prior to performing the imaging studies described. **B.** Incidence of brain signal from MUN14 or M14 after intracardiac (i.c.), intravenous (i.v), or subcutaneous (s.q.) engraftment. **C.** Percentage of animals (n = 10 per group) with bioluminescent signal detected in the brain, spinal cord, kidney, or lungs post-mortem (**p<0.001, ***p<0.0001; Chi-Square analysis) in EBV+ (LCL/B95.8, LCL Mutu, M14, and MUN14) and EBV- B-cells (8226 and BJAB). **D.** MUN14 cells visualized by bioluminescent imaging after i.c. engraftment. Whole body (μCT), brain, brain (with μCT), and spinal cord shown.

To develop a mouse model of EBV-dependent B-cell trafficking to the CNS, we introduced expression vectors for GFP and firefly luciferase into the EBV+ Burkitt Lymphoma (BL) cell line Mutu I (M14) for visualization *in vivo* using the IVIS bioluminescent imaging system (**Fig 1A**). M14 cells were engrafted subcutaneously and then monitored for CNS localization. Initially, we observed rare (3/86) animals with weak luminescent signals in the brain. To enrich for this rare subpopulation, we isolated M14 cells from brains, confirmed GFP positive signal *ex vivo* and then enriched by filtering, sorting for GFP, culturing *in vitro*, and re-engrafting into mice. We repeated this procedure for seven cycles, with the rate of neuroinvasion increasing with each serial passage, resulting in the neuroadaptation of a new EBV+ lymphoma subline termed MUN14 (**Fig 1**).

When M14 and MUN14 cells were engrafted via the intracardiac (i.c.), intravenous (i.v.), or subqutaneous route (s.q.) a higher percentage (**Fig 1B**; n = 10 per group) of cells trafficked to the brain in animals engrafted with MUN14 vs M14, suggesting that MUN14 is more neuroinvasive than M14. To determine if MUN14 is more neuroinvasive than other EBV+ B cell lines, we compared neuroinvasiveness of MUN14 with EBV+ luciferase+ LCLs exogenously transformed with either EBV-B958 (HCLCL) or EBV Mutu I (LCL352) after i.v. engraftment. Again, a significantly higher percentage (p<0.0001, Chi-Square analysis) of animals engrafted with MUN14 showed neuroinvasion (% animals with bioluminescent signal in the brain and spinal cord) compared to other EBV+ and EBV- B-cell lines (**Fig 1C**). Of interest the EBV- multiple myeloma line 8226 was frequently visualized in the joints, jaw, vertebral column, and skull. However, when the brains and spinal cords were removed and imaged at the end of the study, 8226 was only detected in 30% of brains 20% of spinal cords, similar to EBV+ LCLs and M14. Although MUN14 cells could be detected in the kidneys of some animals, MUN14 predominantly homed to the CNS (brain and spinal cord) as demonstrated by μCT and ex vivo imaging of brain and spinal cord (**Fig 1D**).

### Enhanced neuroinvasive and neuroinflammatory properties of the MUN14 B-cell subline

**Fig 2:**
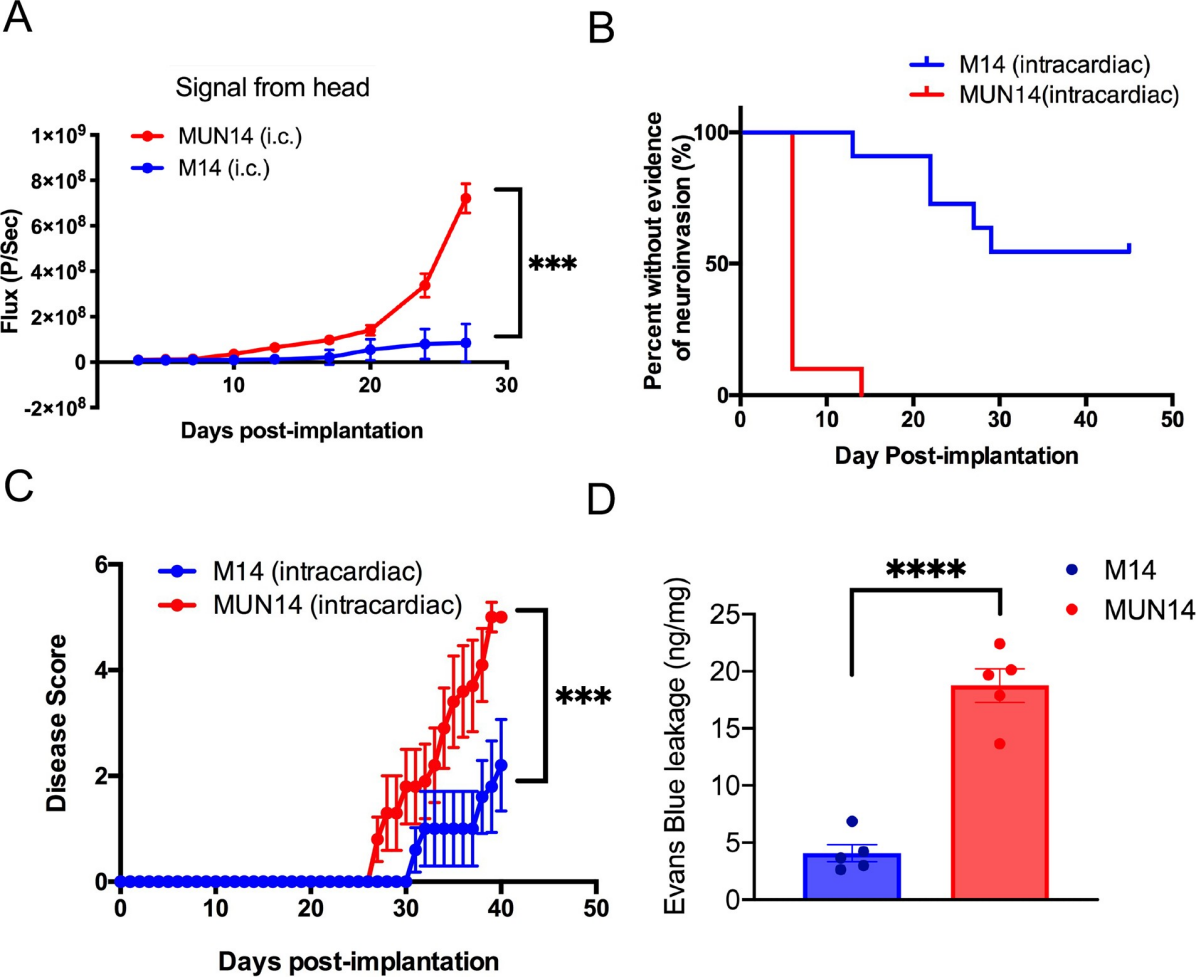
MUN14 is more neuroinvasive than parental M14 line. **A-C.** Animals were engrafted with either MUN14 (red) or M14 (blue) via the intracardiac route. **A.** bioluminescent signal measured by flux in the head (***p<0.0001, Kruskal-Wallis test). **B.** Days until bioluminescent signal was detected in the head of animals engrafted with MUN14 (red) or M14 (blue) (***p<0.0002; log-rank Mantel-Cox test). **C.** Disease score signal in the heads of animals engrafted with MUN14 (***p<0.0002; log-rank Mantel-Cox test). **D.** Evans blue leakage was significantly greater in MUN14 vs M14 engrafted animals 7 days post engraftment (p>0.00001; T Test).

To further examine the neuroinvasive potential of MUN14, we measured the bioluminescent signal in the head of mice engrafted with MUN14 and M14 via the intracardiac route. Bioluminescent imaging using the Spectrum IVIS CT demonstrated significantly increased (***p<0.0001, Kruskal-Wallis test) signal in the heads of animals ingrafted with MUN14 (**Fig 2A**). Moreover, more animals engrafted with MUN14 showed evidence of neuroinvasion and, when neuroinvasion occurred in M14 engrafted animals, it was not detectable until later timepoints (**Fig 2B**). Mice with evidence of EBV+ B cells in the brain showed clear CNS symptoms including seizure, hind limb weakness-shake, gait abnormalities, and paralysis (**S1 Table and S1 Movie**). Moreover, overall disease progression was significantly increased (***p<0.0002; log-rank Mantel-Cox test) in MUN14 engrafted animals. Importantly, the growth rate of M14 and MUN14 in culture and luciferase expression are similar between these two cell lines, indicating that the differences are specifically observed in the CNS (**S1 Fig**). When MUN14 and M14 cells are engrafted directly into the CNS (intracranially), the survival curves in animals engrafted with M14 and MUN14 are similar, suggesting that the cell lines are specifically distinguished by their ability to infiltrate the CNS (**S1 Fig**). When MUN14 cells were engrafted intracerebrally in immunocompetent CD1 mice, they were cleared within five days, suggesting that direct engraftment of EBV+ B cells in the CNS of immunocompetent mice is not feasible for studying EBV-mediated mechanisms of B-cell neuroinvasion and neuroinflammation (**S1 Fig)**.

### Transcriptomic analysis of B-cell neuroinvasion

**Fig 3:**
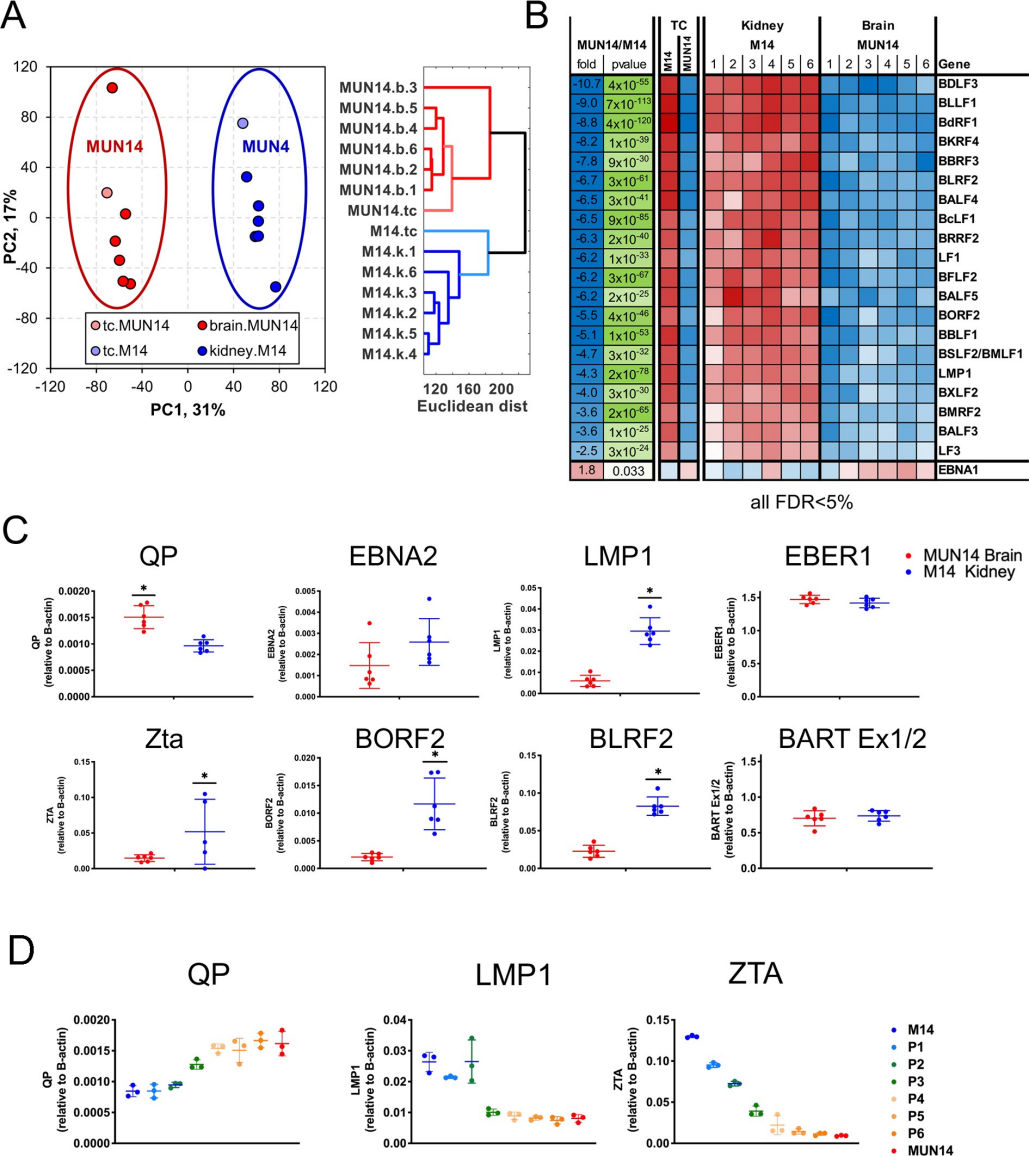
Viral determinants of neuroinvasion identified RNASeq. **A**. Principal component analysis (PCA) of RNA-seq data from MUN14 isolated from brain and in tissue culture compared to M14 isolated from kidney and tissue culture, **B.** Top ten changed EBV genes in MUN14 vs M14. **C.** RT- qPCR of EBV gene expression (EBNA1 QP, EBNA2, LMP1, ZTA, BORF2, and BLRF2) in cells isolated from MUN14 in brain (red) and M14 in kidney (blue) *p<0.005; student t-test. **D.** RT-qPCR analysis of transcripts for Qp, LMP1, and ZTA over serial passaging P1-P6 of M14 in mouse brain. Three mice were assayed for each time point.

To address mechanisms of B cell neuroinvasion and brain penetration, we assayed the transcription profile of the neuroadapted MUN14 using RNA-seq. We compared the differences between MUN14 and M14 in culture as well as MUN14 directly isolated from brain infiltrates and M14 cells directly isolated from the kidney. We chose M14 cells that had kidney infiltrates, because the kidney is the preferred site of colonization for the parental M14 cell line (**Fig 3A**). Principal component analysis (PCA) demonstrated that transcriptional profiles of MUN14 cells isolated from the brains of six animals cluster together with MUN14 in tissue culture and have greater difference when compared to M14 cells isolated from kidney and tissue culture, which are more similar to each other. These data were confirmed by distance plot (**Fig 3B**, false discovery rate< 5%). To identify potential viral determinants of neuroinvasion, we compared expression of EBV genes and found that most viral genes were down-regulated in MUN14, with the exception of EBNA1 that showed a modest relative increase in expression (**Fig 3B**). We confirmed these findings by RT-PCR (**Fig 3C**) and confirmed that expression of EBNA2, LMP1, ZTA, BORF2, and BLRF2 are decreased in MUN14 compared to M14, while expression of the EBNA1 Q promoter (Qp) promoter is elevated in MUN 14. We also found that this change in viral gene expression occurred gradually over the course of neuroadaptation process (**Fig 3D**). This suggests that neuroadapted MUN14 have a restricted EBV latency (type I) compared to the parental M14 cell line showing background relaxed latency (type I/III, leaky) and lytic transcription.

**Fig 4:**
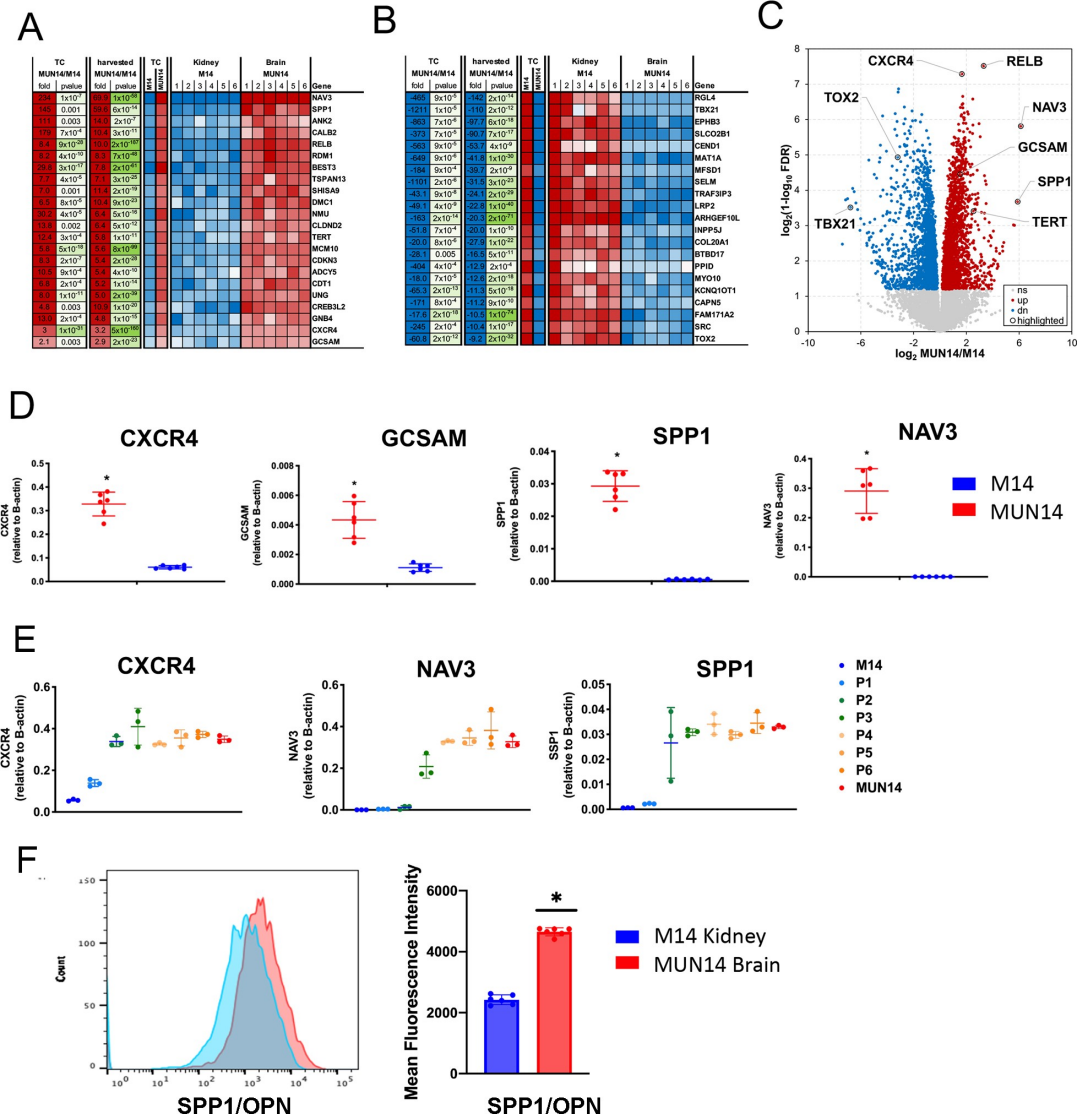
Host determinants of neuroinvasion identified RNASeq. **A**. Top ten changed upregulated host genes in MUN14 vs M14. **B.** Top ten changed downregulated host genes in MUN14 vs M14. **C.** Volcano plot highlighting key upregulated (red) and downregulated (blue) genes in MUN14 vs M14. **D.** RT-q PCR validation of host gene expression (CXCR4, GCSAM, SPP1, NAV3) in cells isolated from MUN14 in brain (red) and M14 in kidney (blue) *p<0.005; student t-test. **E.** RT-qPCR analysis of transcripts for CXCR4, NAV3, and SPP1 over serial passaging P1-P6 of M14 in mouse brain. Three mice were assayed for each time point. **F.** SPP1/OPN cell expression assayed by flow cytometry (using anti-OPN Alexa647).

To identify host cellular determinants of neuroinvasion, we compared expression of host genes. The top upregulated (**Fig 4A**) and top downregulated (**Fig 4B**) are shown. Several genes associated with CNS disease were identified including TSPAN13, RelB, SPP1, and CXCR4 (**Fig 4A**). Volcano plot indicates a similar distribution of up and down regulated genes, with a number of T-cell regulatory genes, such as TBX21 and TOX2 down regulated in brain derived MUN14 (**Fig 4C**). We validated RNA-Seq findings by performing RT-PCR on several genes including CXCR4, Germinal Center associated signaling and motility (GCSAM), secreted phosphoprotein 1 (SPP1), and neuro navigator 3 (NAV3) (**Fig 4D**). We also show that this change occurred gradually across the neuroadaptation process (**Fig 4E**). In addition, these changes in transcription were stable in cells cultured in vitro for 2 months (**S2 Fig**). We confirmed that SPP1/OPN expression was modestly upregulated by flow cytometry in MUN14 compared to M14 cells isolated from the brain and kidney, respectively (**Fig 4F**). These data may not reflect levels of secreted OPN *in vivo*.

### Epigenetic basis for MUN14 neuroadaptation

**Fig 5:**
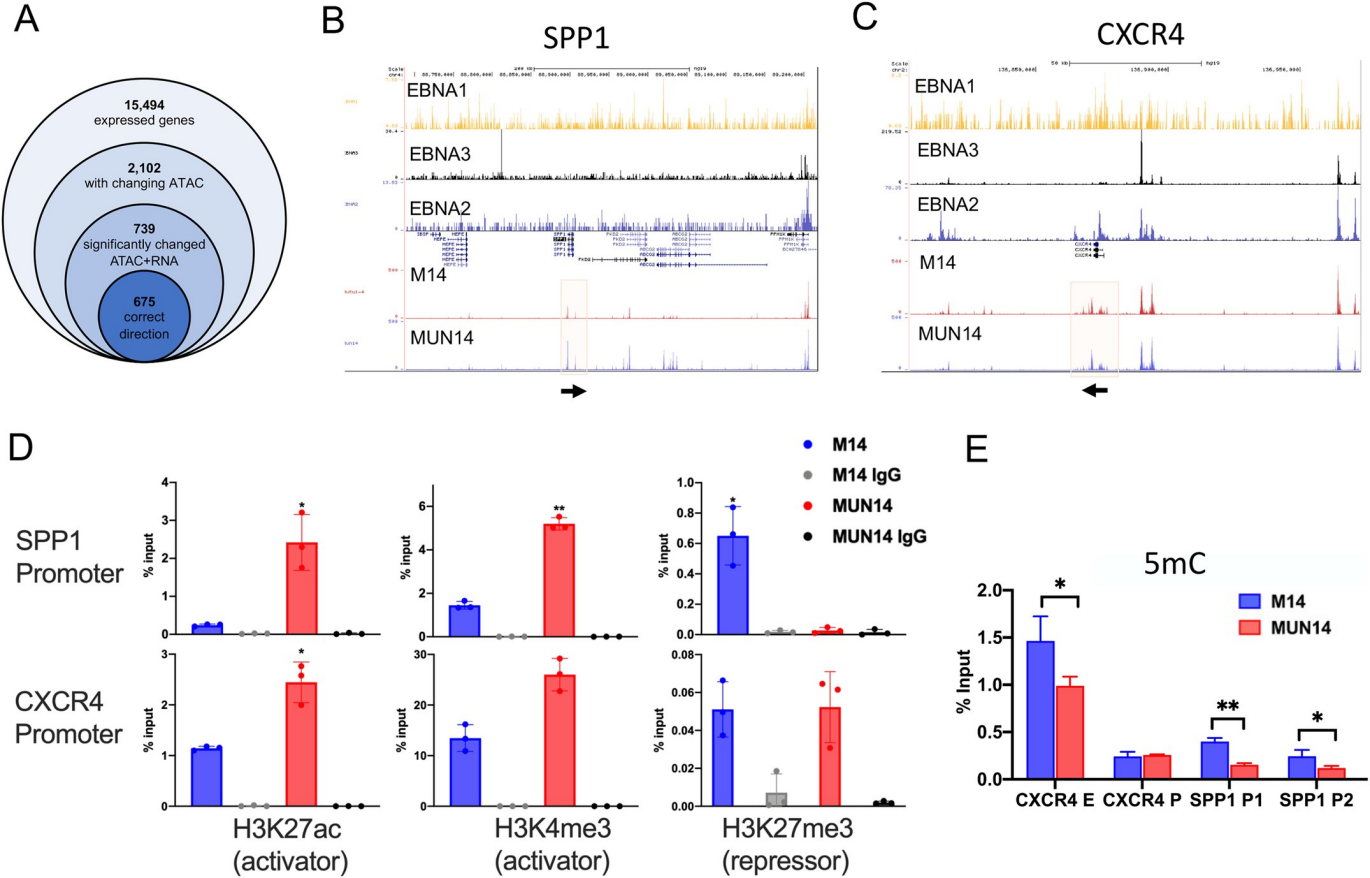
ATAC Seq reveals changes in chromatin accessibility in neuroadapted MUN14. **A.** Transcriptomic data was integrated with ATAC-Seq data by generating sequential subsets of genes based on their association with differentially accessibly chromatin, and the associated gene expression pattern. Genes were considered directly correlated if there was correlation (r > 0.5) between changes in gene expression and changes in chromatin accessibility. The UCSC genome browser was used to map EBNA1, 2, and 3 ChIP-Seq peak files and enrichment beds to the ATAC peaks of M14 and MUN14 for (**B)** SPP1 and (**C)** CXCR4. **D.** ChIP-qPCR of M14 (blue) and MUN14 (red) cells was performed for H3K27ac, HeK4me3, and H3K27me3 using antibodies for the SPP1 and CXCR4 promoters. **E.** MeDIP of promoters of SSP1 and CXCR 4 (bottom).

To determine whether epigenetic mechanisms account for differential expression of key genes, we performed ATAC-Seq on MUN14 and M14 cells. To better define the relationship between the ATAC-Seq and transcriptomic datasets, we identified 2102 genes that had both significant changes in RNA expression and ATAC signal, and 739 of those showed a correlation (Pearson r > 0.5) between ATAC-Seq signal pattern and RNA expression pattern. The areas of differentially accessible chromatin associated with these 675 genes, referred to as the directly correlated genes, as defined by the GeneHancer database [62]. We observed quantitative and qualitative changes in ATAC-seq peaks at several of the differentially regulated genes, including SPP1 and CXCR4 (**Fig 5B and 5C**). We also observed EBV transcription factor binding at CXCR4 and SPP1 gene regulatory loci **(Fig 5B and 5C**). Changing ATAC-seq peaks overlapped with EBNA2 and EBNA3C ChIP-Seq peaks for CXCR4 gene locus (**Fig 5C)**. ATAC-Seq peaks increased at the SPP1 transcriptional start site in MUN14 cells, but these did not correspond to EBNA1, 2 or 3C ChIP-seq (**Fig 5B**), suggesting that cellular factors predominantly regulate SPP1 upregulation. To determine if histone modifications may account for some of the changes in gene expression at the CXCR4 and SPP1 genes, we assayed this region by ChIP-qPCR with antibodies for H3K27ac (activator), H3Kme3 (activator), and H3k27me3 (repressor). We found that SPP1 and CXR4 promoter regions had enriched activation marks for H3K27ac and H3K4me3 in the MUN14 cell relative to M14, correlating with increase in transcription (**Fig 5D**). Interestingly we found that SPP1 promoter has differentially elevated repressive mark for H3K27me3 in M14 relative to MUN14, again correlating with transcriptional differences. Taken together, these findings indicate that changes in neuroadapted MUN14 gene expression correlate with epigenetic modifications at these gene regulatory elements, some of which are directly bound by viral transcription factors.

To further evaluate epigenetic modifications of EBV gene expression in MUN14, we investigated CpG methylation of key EBV promoters of lytic infection and latency by Methylcytosine-DNA immunoprecipitation (MeDIP) using real-time PCR amplimers for the CXCR4 promoter and enhancer as well as two promoter sites for SSP1 (**Fig 5E**). We observed decreased methylation of the CXCR4 enhancer and two SSP1 promoter regions in MUN14 compared to M14. These data suggest that differences in the epigenetic regulation of CXCR4 and SSP1 in these cell lines is consistent with the increased expression of CXCR4 and SSP1/OPN observed in MUN14.

### SPP1/OPN is functionally important for B-cell neuroinvasion

**Fig 6:**
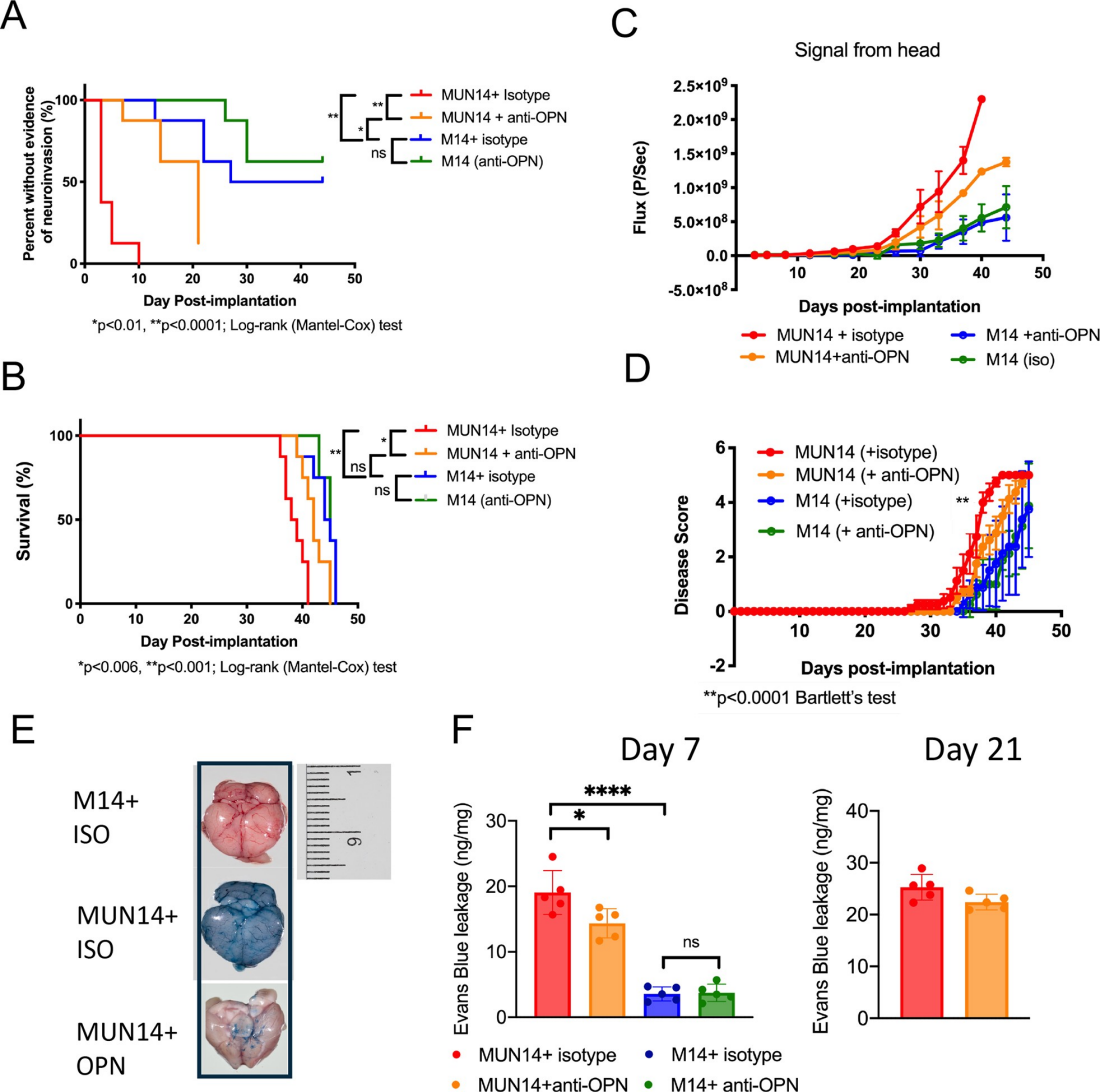
Neuroinvasion of MUN14 is attenuated with an osteopontin-specific neutralizing antibody. **A-F.** Animals were engrafted i.c. with MUN14 and treated with isotype (red) or anti-OPN (orange) or engrafted with M14 and treated with isotype (blue) or anti-OPN (green). N = 10 animals (5 female and 5 male) per group. **A.** Days until bioluminescent signal was detected in the head. **B**. bioluminescent signal measured by flux in the head. **C.** Survival curve (*P<0.006, **P<0.001; Log-rank (Mantel-Cox) test) **D**. Disease score (**p<0.0001; Bartlett’s test). **E.** Evans Blue stain. **F.** Quantitation of Evans blue stain.

To determine if differentially regulated genes contribute functionally to neuroinvasion of EBV+ B cells, we focused on the SPP1/OPN gene because it has well-defined neutralizing antibodies to its protein product that can be used in animal studies. We therefore treated NSG mice with a neutralizing antibody to OPN or isotype control and then engrafted with MUN14 or M14 and treated animals i.p. Subsequent to engraftment, animals were treated every three days with anti-OPN or isotype control. We found that anti-OPN antibodies delayed time to neuroinvasion (**Fig 6A**), increased survival (**Fig 6B**) and decreased the growth of cells in the CNS (**Fig 6C**) compared to MUN14 isotype treated control animals. In addition, the clinical disease course was significantly improved in MUN14 engrafted animals treated with anti-OPN (**Fig 6D**). Evans blue staining, revealed widespread blood brain barrier penetrability in MUN14 engrafted animals which is also reduced by treatment with anti-OPN (**Fig 6E and 6F**). These findings indicate that OPN/Spp1 contributes to the the neuroinvasive phenotype of EBV+ B-cells, but additional host factors must also contribute to the full neuroinvasive and CNS growth phenotype of MUN14 cells.

## Discussion

Immune cell trafficking across endothelial barriers is critical in maintaining appropriate immune responses in the brain and circulating B cells routinely enter the CNS as part of normal immune surveillance [63]. However, dysregulated trafficking of pathogenic B cells has been implicated in the pathophysiology of several disorders of the CNS including PCNSL and MS [64,65]. Therefore, it is critical to better understand molecular mechanisms that influence B cell interactions with the blood brain barrier and mediate B cell neuroinvasion. Here, we have found that EBV+ BL cell line can undergo an epigenetic switch to become more neuroinvasive. We identified differentially regulated genes, including CXCR4 and SPP1, that undergo epigenetic changes at the gene loci that correlates with transcriptional changes. We show that antibody to SPP1 reduces CNS trafficking and neuroinvasion.

Host and viral factors that direct B cells to the CNS are unclear. In our murine model of CNS neuroinvasion of EBV+ lymphoma cells, RNA-Seq analysis of MUN14 cells isolated from the brain revealed that this neuroadapted line overexpresses a number of host factors that are known to be upregulated in PCNSL, including the stromal cell derived factor-1 (SD-1)-CXCR4 signaling pathway, TSPAN13, germinal center signaling and motility protein (GCSAM), and OPN/SSP1, [66–69] (Fig 4A). OPN is an extracellular matrix protein with protean physiological functions and is known to be involved in bone remodeling, cancer, wound healing, and a number of inflammatory diseases [70,71]. It is expressed in immune cells including T cells, B cells dendritic cells, and macrophages and contributes to inflammation and induction of autoimmunity via increased expression of proinflammatory cytokines, and inhibition of IL-10 [72,73]. Notably, osteopontin is uniformly expressed in PCNSL with one study [74] reporting 100% (20/20) positivity for osteopontin expression in PCNSL tumors by immunohistochemistry, suggesting that the increase in osteopontin expression in MUN14 is consistent with reported neuropathological findings in PCNSL.

**Fig 7:**
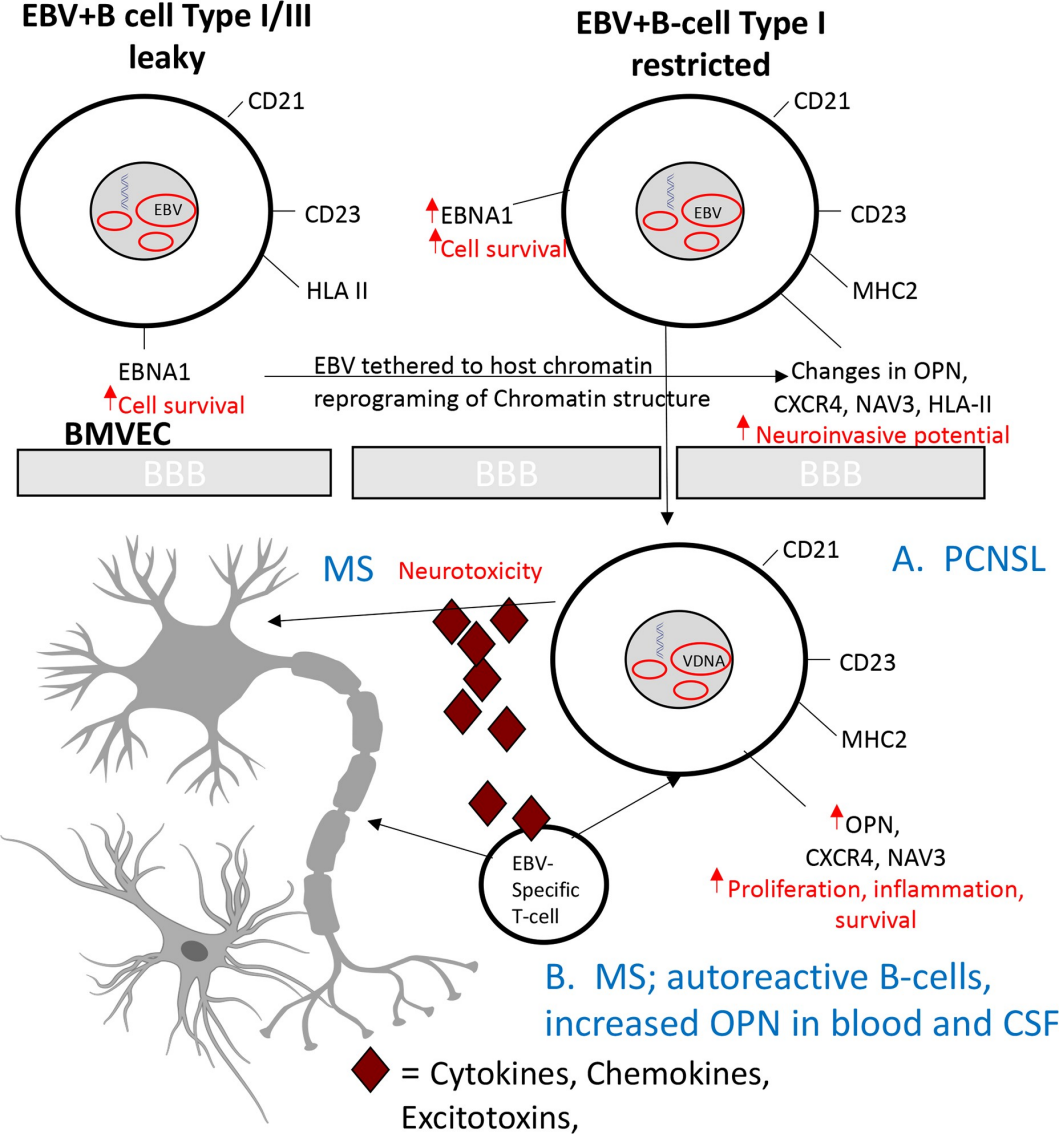
Theoretical Model: Epigenetic reprogramming of EBV+ B-cell contributes to neuroinvasion and CNS disease. Epigenetic changes that alter the expression of viral and host genes in EBV+ B cells, resulting in a neuroinvasive phenotype with increase expression of SPP1/OPN, CXCR4, and NAV3. We speculate that increased neuroinvasion of B-cells with oncogenic potential leads to PCNSL (**A**), while the infiltration of autoreactive and inflammatory B-cells may contribute to the neuropathology of MS (**B**).

Importantly, the ability to inhibit the neuroinvasion of MUN14 cells with a neutralizing antibody to OPN/SPP1 suggests that overexpression of osteopontin in PCNSL contributes to the pathogenesis of this disorder, by increasing the propensity of these cells to enter the CNS (**Figs 6 and 7**). It has long been appreciated that increased OPN expression is associated with increased autoantibody secretion and, in the case of Sjögren’s disease, increased lymphocytic infiltration into salivary tissue—the target of the autoimmune response in that disorder [75,76] [76].Therefore, osteopontin may be involved in the CNS invasion of other pathogenic and autoreactive B-cells into the CNS and contribute to the neuropathogenesis of CNS inflammatory disorders, including MS. OPN gene expression is increased in MS brain lesions [77] and variants of the OPN gene influence the risk of developing MS as well as serum OPN levels, [77,78]. In addition, T cells from MS patients and in mouse models of EAE have increased OPN receptors [79]. OPN levels have been correlated with increased disease progression and recurrent relapses [80,81] and both CNS and blood concentrations of OPN have been shown to have diagnostic and prognostic value in MS [82]. Moreover, in EAE, OPN deficiency is associated with resistance to progressive EAE and frequent disease remissions [83,84]. Our study suggests that, in addition to its role in inducing inflammation and promoting T cell differentiation in MS, OPN likely plays an important role in facilitating the neuroinvasion of EBV+ and potentially, autoreactive B cells (Fig 7). Further, the increased expression of EBNA1 and apparent restriction of EBV latency suggests that EBV/EBNA1 may drive the observed epigenetic changes by tethering to host chromatin, thereby promoting global reprogramming of chromatin structure and cellular transcription [85,86] [87] (Fig 7). Indeed, recent 4C analysis from our laboratory has demonstrated that EBV tethering sites vary according to latency type and that strong EBV tethering sites are highly enriched for neuronal genes [88]. Tethering of EBV to chromosome regions containing NRNX1, NAV 3, and SYTI leads to their transcriptional repression. Therefore, the increased expression of NAV 3 expression observed in MUN14 may suggest the use of alternative tethering sites for EBV in this neuroadapted cell line.

In addition to known drivers of cell adhesion and migration, several transcription factors, including T- box transcription factor TBX21 (TBET) and Tox2 were differentially expressed in the neuroadapted MUN14 line compared to the parental M14. TBET is predominantly known for its role in T cell development. In B cells, TBET promotes IFN-γ production and enables class switching to IGg2a [89] [90]. TBET+ plasmablasts are associated with immune exhaustion and have been implicated in autoimmune diseases, including Sjögrens syndrome, systemic lupus erythematosus, MS, and in relevant animal models [91,92]. TBET has also been shown to be upregulated in regulatory B-cells that can down regulate T-cell activity [92]. In addition, TBET expression in B cells is required for IgG2a/c class switching and promotes migration of B cells to the centroblast-rich dark zones of germinal centers, which are proximal to T cell areas [93]. Of interest TBET dependent expression of osteopontin in T cells is important in driving T cells toward a Th1, proinflamatory phenotype [94]. Future studies will be required to investigate the functional role of TBET and Tox2 in B-cell regulation and CNS trafficking.

Notably, neuron navigator 3 (NAV3) is highly and stably (Figs 4A and S2) upregulated in MUN14 cells. NAV3 belongs to a group of proteins that bind microtubles and facilitate cytoskeleton reorganization and induce neurite like extensions [95,96]. Nav3 is aberrantly expressed in diverse tumors and has been shown to direct migration and dissemination of cancer cells. It has been suggested that NAV3 amplifications may improve prognosis for some CNS tumors [97]. The upregulation of NAV3 in the MUN14 model indicates that this gene may also play a role in the migration of pathogenic B-cells to the CNS; this putative relationship requires further exploration.

Inflammatory B cells are thought to contribute to the pathophysiology of MS both in the periphery and in the CNS [98–102]. More recently, it has been demonstrated that the clonal expansion of inflammatory memory and plasma cells in the CSF is associated with inflammation, blood-brain barrier breakdown, and oligoclonal bands/intrathecal Ig synthesis [103]. Therefore, limiting the migration of B cells into the CNS represents an important therapeutic strategy in MS as supported by the therapeutic success of monoclonal antibody therapies against CD20-expressing B cells and the cell adhesion molecule a4-integrin (Natalizumab) as well as the synthetic purine analogue Cladribine, which is used to target B-cells in B-cell chronic lymphocytic leukemia and MS [104]. The development of new therapies targeting neuroinvasive B cells may provide more selective and effective treatments for MS. The MUN14 model represents a new tool for identifying factors that contribute to the migration of oncogenic/autoreactive B-cells to the CNS and for testing potential treatment modalities, including epigenetic modulators, that prevent development of a B-cell neuroinvasive phenotype.

## Materials and methods

### Ethics statement

All animal experiments were conducted under The Wistar Institute’s approved Institutional Animal Care and Use Committee Protocol #201161 in accordance with the Committee for the Purpose of Control and Supervision of Experiments on Animals guidelines for animal experimentation. All mice in this study were managed in accordance with the NIH Office of Laboratory Animal Welfare: “PHS Policy on the Humane Care and Use of Research Animals” and the recommendations of the American Association for Accreditation of Laboratory Animal Care (AAALAC).

### Mice

NSG mice (NOD.Cg-*Prkdc*^*scid*^ *Il2rg*^*tm1Wjl*^/SzJ) were bred in-house at The Wistar Institute under protocol #112092 and maintained in a designated, specific pathogen-free environment. Mice were enrolled at 8 weeks of age and fed sterile food and water *ad libitum*. Upon reaching a humane endpoint or termination of the study, mice were euthanized according to AALAC euthanasia guidelines via CO_2_ administration.

### Engraftment and imaging studies

We previously developed an EBV^+^ B cell lymphoma model, using cells (the EBV^+^ cell line Mutu I) transduced to express GFP and firefly luciferase, referred to as M14 [105]. We observed rare CNS-colonies of M14 cells when these cells were engrafted subcutaneously. The CNS-colonies of EBV+ B cells were then isolated and transplanted subcutaneously and found to have high frequency of brain infiltration by bioluminescent imaging using the Spectrum IVIS CT (Perkin-Elmer; Waltham, MA). Serial transplanting increased the frequency of neuroinvasion, and the generation of new EBV^+^ B lymphoma subline, referred to as MUN14. Microsattelite testing was performed to confirm that the serially passaged MUN14 line was indeed derived from M14 (**S1 Fig**).

5x10^5^ MUN14 or M14 cells were engrafted into NSG mice (5 females and 5 males per group) by the subcutaneous (s.q.), intravenous (i.v.), intracranial, or intracardiac (i.c.) routes. In addition, LCLs derived from peripheral blood mononuclear cells from healthy controls that were exogenously transformed with EBV-B958 (NHCLCL) or EBV-Mutu (LCL352) were engrafted via the i.v. route. Mice were imaged using the IVIS Spectrum CT two days after engraftment and twice per week thereafter. Full body images and head-only images were acquired. Head-only images were acquired using a 3D printed device (Original Prusa i3 MK3S) using a 1.75 mm Hatchbox polylactic acid filament (see **S1 Fig**) to prevent masking of signal in the brain from the periphery so that early timepoints of neuroinvasion could be visualized and quantified. For imaging studies, mice were injected with D-luciferin (Gold Biotechnology), i.p. at a dose of 7.5 mg/kg in a dose volume of 10 ml/kg body weight 15 minutes prior to imaging; this was the optimal interval between luciferin injection and bioluminescent imaging as determined by an initial kinetic curve for these cell lines in mice. Mice were anesthetized using isoflurane prior to imaging. Total body Flux (Photons/Sec) and Flux from the head only were quantitated throughout the study using the IVIS imaging software. Upon necropsy, brains, liver, lungs, kidneys, and spinal cords were imaged in the IVIS Spectrum CT (**Fig 1D**) to confirm the presence of EBV+ B cells.

Mice were weighed every other day and scored neurological signs (0 = healthy; 1 = lethargy and inactivity; 2 = weight loss; 3 = limb-shake or weakness; 4 = hind limb paralysis; 5 = moribund and death). Death was not an endpoint and animals were sacrificed for significant cachexia (including greater than 20% body weight) or if they reached clinical stage 4.

### Evans blue stain

Mice were injected i.v. with Evans blue stain (Sigma Chemical Co., Saint Lois MO) at 2% wt/vol in PBS (3 ml/kg) into the mouse tail vein and 1.5 h after the mice were perfused with saline (250 ml) through the left ventricle until colorless perfusion fluid was obtained from the right atrium. The brain tissue was isolated and homogenized in 3 ml of N,N-dimethylformamide, incubated for 18 h at 55°C, and centrifuged (10,000 rpm) for 20 minutes. The supernatant fluorescence was analyzed using a spectrophotometric quantification of BBB permeability was calculated by measuring the content of Evans blue in the brain tissue from a linear standard curve derived from known amounts of the dye and its fluorescence intensity and is expressed as mg/ml of brain tissues.

### Treatment with Osteopontin (OPN) Neutralizing Antibody

Mice were dosed with either anti-OPN (clone MPIIIB10, DSHB, University of Iowa) or isotype control (Biocell) at a concentration of 100μg/mouse per injection, i.p. one day prior to intracardiac engraftment of 5x10^5^ MUN14 or M14 cells and again every three days after engraftment.

### RNASeq

MUN14 and M14 cells obtained from the brains and kidneys of mice were filtered through a 40μM and then a 30μM filter prior to sorting for GFP to isolate MUN14 or M14 cells from the brain or kidney. Total RNA was extracted using a QiaShredder followed by RNA isolation with the RNeasy kit (Qiagen).

RNA samples were submitted to the Wistar Institute genomics core facility for initial analysis of RNA quality, with each sample having a RIN value greater than 8.5 (TapeStation, Agilent Technologies). Sequencing library preparation was then completed using the QuantSeq 3’-mRNA kit (Lexogen) to generate Illumina-compatible sequencing libraries according to the manufacturer’s instructions. Sequencing was done with an Illumina NextSeq500 on high output mode to generate ~4x10^8^ 75bp reads across 8 multiplexed and pooled samples.

### ATAC SEQ

ATAC-seq was performed in three biological replicates according to the Omni-ATAC-seq protocol with modifications. Briefly, 1 × 10^5^ cells (>95% viability) were washed in 50 μl cold PBS, spun down at 500 x g and 4°C for 5min, and resuspended in 50 μl cold ATAC-Resuspension Buffer (RSB) (10 mM Tris-HCl, pH7.4, 10 mM NaCl, 3 mM MgCl_2_) containing 0.1% IGEPAL CA-630, 0.1% Tween-20 and 0.01% Digitonin. Resuspended cells were kept on ice for 3 min, then washed with 1 ml cold ATAC-RSB containing 0.1% Tween-20 but no IGEPAL CA-630 or Digitonin. Pellet nuclei at 500 x g and 4°C for 10 min, and the supernatant was removed. The pellet was then resuspended in a 50 μl Tn5 transposase reaction mixture following the manufacturer’s protocol (Nextera Tn5 transposase kit, Illumina) and incubated at 37°C for 30 min in a thermomixer with 300 rpm mixing. DNA was purified using a MinElute PCR purification kit (Qiagen) and eluted in 10 μl Elution Buffer for library amplification. PCR amplification of tagmented DNA was done using the NEBNext HiFi PCR mastermix (New England Biolabs) with a universal forward and sample-specific reverse oligo for sample barcoding using the following PCR conditions: initial incubations of 72°C for 5 min and 98°C for 30 s, followed by 5 cycles of 98°C for 10 s, 63°C for 30 s, and 72°C for 1 min. Additional number of cycles was determined for each sample through a “side” qPCR reaction using an aliquot of the PCR as template to determine the number of cycles needed to reach 1/3 of the max fluorescence. Total cycle numbers ranged from 8 to 9 cycles. PCR products were run on a 1% agarose gel, regions from ~50 bp to ~1 kb were excised, and DNA was extracted using a gel extraction kit (Qiagen). Purified DNA was submitted to the Wistar Institute Genomics core facility for quality analysis and sequencing. All samples were sequenced on NextSeq500 (Illumina) to generate paired-end 2x42 bp reads.

### RT-qPCR

RNA was isolated from 2 x 10^6^ cells using RNeasy Kit (Qiagen) and then further treated with DNase I by using DNase treatment and removal kit (Ambion). Real-time PCR was performed with SYBR green probe in an ABI Prism 7900 and the delta Ct method for relative quantitation. Primer sequences for RT-PCR are available upon request.

### ChIP-qPCR

Cells were crosslinked in 1% formaldehyde for 15 min, followed by quenching for 5 min with 0.125 M glycine. 1 × 10^7^ cells were lysed in 1 ml SDS lysis buffer (1% SDS, 10 mM EDTA, and 50 mM Tris-HCl, pH 8.0) containing 1 mM PMSF and protease inhibitor cocktails (Sigma-Aldrich), and kept on ice for 10 min. Then lysates were sonicated with a Diagenode Bioruptor, cleared by centrifugation to remove insoluble materials, and diluted 10 fold into IP Buffer (0.01% SDS, 1.1% Triton X-100, 1.2mM EDTA, 16.7mM Tris pH 8.0, 167mM NaCl, 1 mM PMSF, and protease inhibitors cocktail), and incubated with anti-H3K27ac (Abcam, ab4729), anti-H3K4me3 (Millipore, 07473), or anti-H3K27me3 (Active motif, 39155) overnight at 4°C. Preblocked protein A sepharose (GE Healthcare, 17-0780-01) was added to each IP reaction for additional 2 to 3 h incubation at 4°C. Each immune complex was washed five times (10 mins each) in ChIP related wash buffer at 4°C, and eluted with 150 μl Elution buffer (10mM Tris, pH 8.0, 5mM EDTA, and 1% SDS) at 65°C for 30 min. The elutes were then incubated at 65°C overnight to reverse cross-linking, and further treated with Proteinase K in a final concentration of 100 μg/ml at 50°C for 2 hrs. ChIP DNA was purified by Quick PCR Purification Kit (Life Technologies) following the manufacturer’s instruction. ChIP DNA was assayed by qPCR using primers specific for indicated regions and quantified as % input.

**Methylcytosine-DNA immunoprecipitation (MeDIP)** was performed as described previously. Genomic DNA was purified using Wizard Genomic DNA Purification Kit (Promega, A1120), sheared into 100–500 bp fragments using Bioruptor (Diagenode), then subjected to methylcytosine-DNA-immunoprecipitiation (MeDIP) assays using MagMeDIP kit (Diagenode, C02010021). Quantification of precipitated DNA was determined using real-time PCR and the delta Ct method for relative quantitation.

## Supporting information

S1 Fig MUN14 does not have an accelerated disease course compared to M14 when cells are engrafted via the intracerebral route. Animals were engrafted with either MUN14 (red) or M14 (blue) via the intracerebral route. **A.** There is no difference in survival of NSG mice engrafted directly into the brain with M14 vs Mun14 **B.** Growth curves, measured by bioluminescent signal from the head, are similar in M14 vs MUN14. MUN14 signal is higher (p = 0.05; Mann-Whitney) at the final timepoint. **C.** CD1 mice reject MUN14 cells within 5 days post intracerebral engraftment of MUN14 cells. Head-only images were acquired using a 3D printed device to prevent masking of signal in the brain from the periphery for high sensitivity measuring of the head.
(TIF)

S2 Fig Transcriptional and epigenetic changes in MUN14. Epigenetic changes in NAV3 and SPP1/OPN are stable. RT-PCR performed after MUN14 cells were cultured for three-months ex vivo (from mouse brain) demonstrates that epigenetic changes leading to increased expression of NAV3 and SPP1 are highly stable, while other changes in expression (e.g.CXCR4) are not sustained with prolonged culture outside of the CNS microenvironment.
(TIF)

S1 Table Neurological Features of mice engrafted with MUN14 via either the intracranial, intracardiac, or intravenous route. Mice injected with MUN14 cells through intracranial (column 1) or intracardiac or intravenous (column 2) were scored for neurological dysfunctions: (1) rapid loss of weight, (2) seizures, (3) hydrocephalus, (4) hind limb weakness, (5) complete hind limb paralysis, (6) urinary incontinence, (7) circling, (8) axial rotary spin/roll, (9) tremors. Check mark indicates phenotype observed. X marks not observed.
(TIF)

S1 Movie Circling behavior in mice with evidence of brain infiltration of MUN14 cells by IVIS bioluminescent imaging. (MOV)

S2 Movie Hind limb paralysis in mice with evidence of brain infiltration of MUN14 cells by IVIS bioluminescent imaging. (MOV)
