# Supplementary figures and images for: Epigenetic Plasticity Enables CNS-Trafficking of EBV-infected B Lymphocytes
Source: PLoS Pathog. 2021 Jun 9;17(6):e1009618. doi: 10.1371/journal.ppat.1009618 (PMC8216538; doi:10.1371/journal.ppat.1009618)

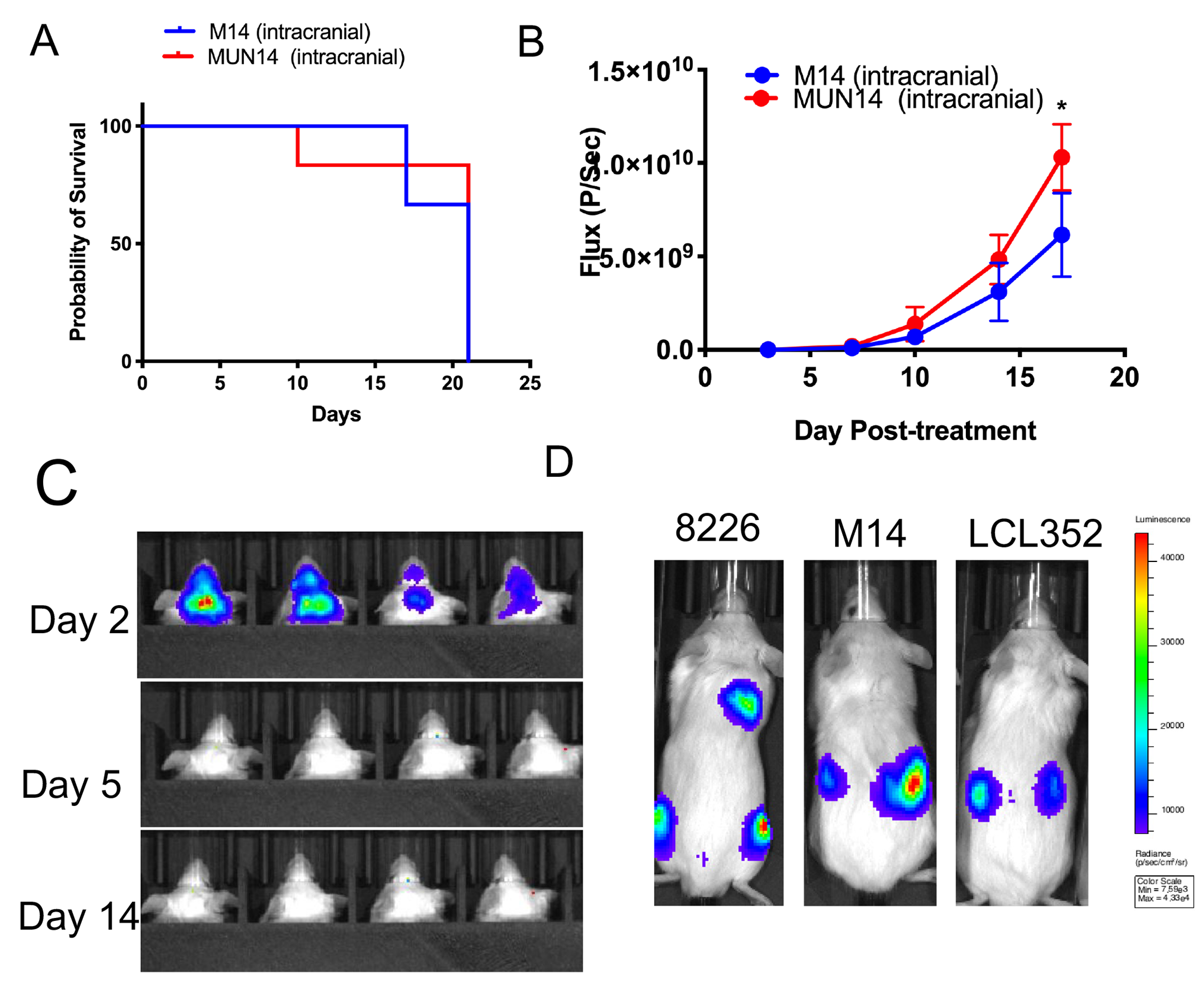

Supplement: S1 Fig — Animals were engrafted with either MUN14 (red) or M14 (blue) via the intracerebral route. A. There is no difference in survival of NSG mice engrafted directly into the brain with M14 vs Mun14 B. Growth curves, measured by bioluminescent signal from the head, are similar in M14 vs MUN14. MUN14 signal is higher (p = 0.05; Mann-Whitney) at the final timepoint. C. CD1 mice reject MUN14 cells within 5 days post intracerebral engraftment of MUN14 cells. Head-only images were acquired using a 3D printed device to prevent masking of signal in the brain from the periphery for high sensitivity measuring of the head. (TIF) [file ppat.1009618.s001.tif]

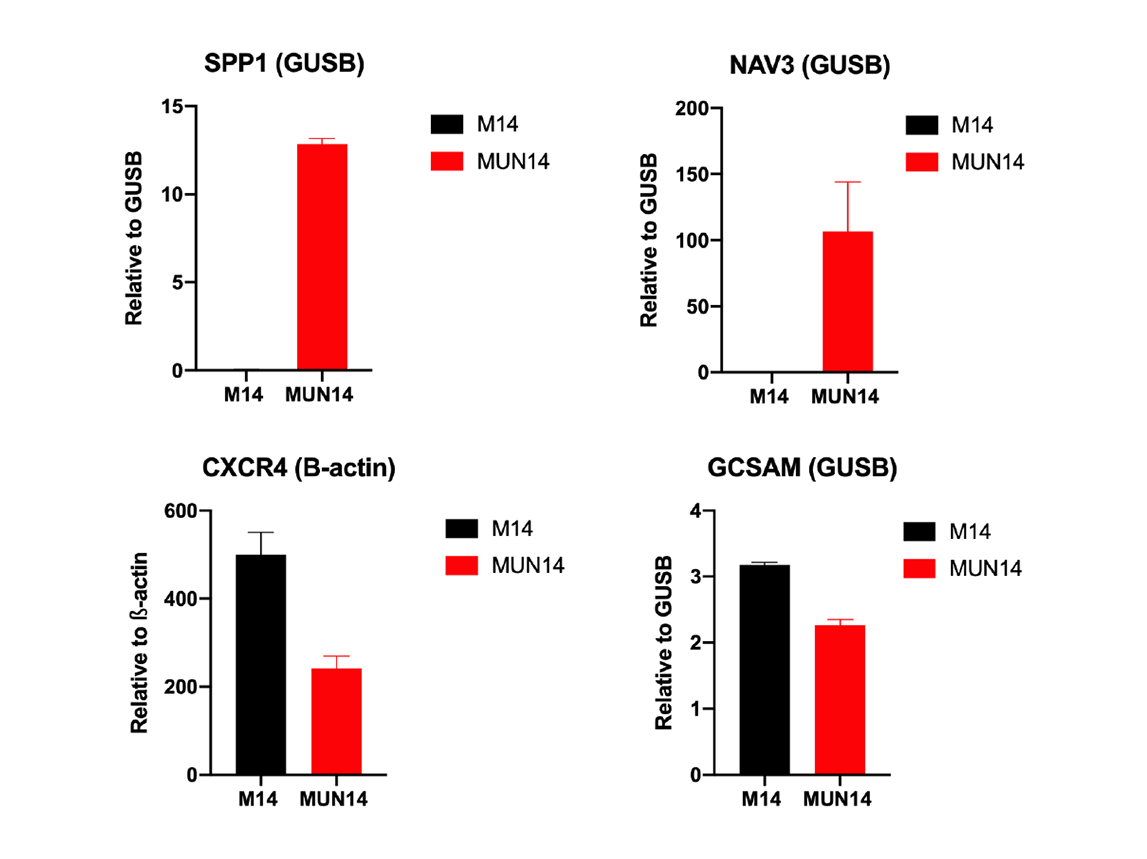

Supplement: S2 Fig — Epigenetic changes in NAV3 and SPP1/OPN are stable. RT-PCR performed after MUN14 cells were cultured for three-months ex vivo (from mouse brain) demonstrates that epigenetic changes leading to increased expression of NAV3 and SPP1 are highly stable, while other changes in expression (e.g.CXCR4) are not sustained with prolonged culture outside of the CNS microenvironment. (TIF) [file ppat.1009618.s002.tif]

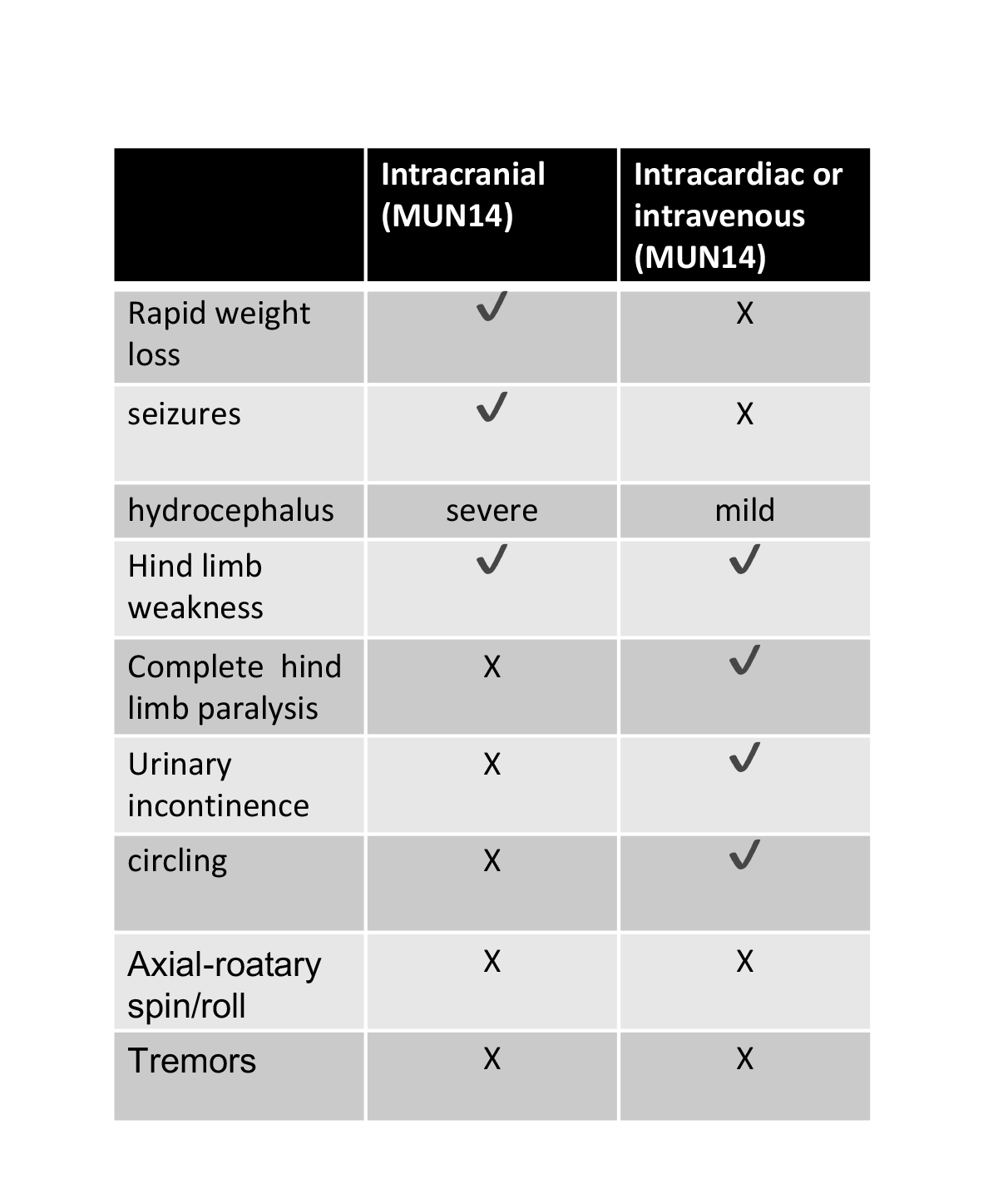

Supplement: S1 Table — Mice injected with MUN14 cells through intracranial (column 1) or intracardiac or intravenous (column 2) were scored for neurological dysfunctions: (1) rapid loss of weight, (2) seizures, (3) hydrocephalus, (4) hind limb weakness, (5) complete hind limb paralysis, (6) urinary incontinence, (7) circling, (8) axial rotary spin/roll, (9) tremors. Check mark indicates phenotype observed. X marks not observed. (TIF) [file ppat.1009618.s003.tif]
